# HDAC11 inhibition disrupts porcine oocyte meiosis via regulating α-tubulin acetylation and histone modifications

**DOI:** 10.18632/aging.202697

**Published:** 2021-03-19

**Authors:** Rong Huang, Liyan Sui, Cong Fu, Yanhui Zhai, Xiangpeng Dai, Sheng Zhang, Ziyi Li

**Affiliations:** 1 Key Laboratory of Organ Regeneration and Transplantation of Ministry of Education, First Hospital, Jilin University, Changchun 130021, Jilin, China

**Keywords:** oocyte, meiosis, HDAC11, α-tubulin acetylation, histone H4 acetylation

## Abstract

HDAC11, the sole member of HDAC class IV family, plays vital roles in activating mitosis and apoptosis of tumor cells, but its functions in meiosis are rarely investigated. In the present study, the effect of HDAC11 on meiosis during porcine oocytes maturation was fully studied. The results showed that HDAC11 inhibition by its specific inhibitor JB-3-22 dramatically decreased the porcine oocyte maturation rate by disturbing spindle organization and chromosomes alignment without affecting the cytoplasmic maturation. Further study indicated that HDAC11 inhibition significantly elevated the acetylation levels of α-tubulin and H4K16, which are crucial for spindle organization and chromosomes alignment. Moreover, immunofluorescence staining results showed that HDAC11 inhibition also disturbed other meiosis-related histone modifications, such as increased H3S10pho, H4K5ac and H4K12ac levels and reduced H3T3pho level. Furthermore, RNA-seq analysis results indicated that HDAC11 inhibition disturbed porcine oocytes transcriptome (157 up-regulation, 106 down-regulation). In addition, HDAC11 inhibition compromised oocytes quality and subsequent development after parthenogenetic activation, which may be caused by the aberrant nuclear maturation and transcriptome expression profile during oocytes maturation. Therefore, our results elucidate the function of HDAC11 in porcine oocytes maturation and embryos development through regulating α-tubulin acetylation, meiosis-related histone modifications and transcriptome.

## INTRODUCTION

Chromosome segregation errors which could cause spontaneous abortions, birth defects and congenital defects in humans, frequently happen during the meiosis [1]. It was reported that many biological events, such as spindle organization, spindle assembly checkpoint (SAC), the attachment between kinetochores and microtubules (K-MT), ensured the correct chromosome segregation during the process of meiosis. However, some epigenetic modification of histone or non-histone proteins during meiosis could affect the chromosome segregation and subsequently decrease the oocyte quality.

Interestingly, several HDACs, such as HDAC2 [2], HDAC3 [3], HDAC6 [4] and HDAC8 [5], have been reported to be necessary for spindle organization, SAC, the attachment between kinetochores and microtubules (K-MT) during oocytes meiosis through regulating α-tubulin acetylation, γ-tubulin location and H4K16 acetylation. Therefore, histone deacetylation mediated by histone deacetylases (HDACs) plays a vital role in oocyte maturation [6]. The maturation-associated aberrant of histone acetylation frequently happened due to disturbed HDAC activity or histone acetyltransferases (HATs) activity [7].

HDACs are divided into four classes according to the homology, subcellular localization and enzyme activity, namely class I (HDACs 1,2,3,8), class IIa (HDACs 4,5,7,9), class IIb (HDACs 6,10), class III (Sirtuins 1-7) and IV (HDAC11) [8, 9]. In addition to class III, which requires nicotinamide adenine dinucleotide (NAD+) as an essential cofactor to function, the other three classes are Zn^2+^-dependent enzymes, also known as classical deacetylases [10]. HDAC11 is the only member of class IV HDAC family. It is highly expressed in the brain, heart, skeletal muscle, kidney and testis in humans [11]. Previous study indicated that HDAC11 blockage could induce apoptosis of tumor cells in neuroblastoma via preventing spindle assembly and cell cycle [12], but the exact role of HDAC11 in meiosis is largely unknown.

In view of sequence similarity of catalytic core region of HDAC11 with class I and II HDACs at amino acid level [8], we found the expression of HDAC11 during oocytes meiosis. Therefore, in this study, we sought to explore the critical roles of HDAC11 in oocytes maturation [13, 14]. Interestingly, our findings reveal that HDAC11 inhibition disturbs α-tubulin acetylation, meiosis-related histone modifications (H3T3pho, H3S10pho, H4K5ac, H4K12ac and H4K16ac) and transcriptome profile, and subsequently causes aberrant oocytes maturation and embryos development. This study provides an insight into the roles of HDAC11 in exploring the underlying mechanisms for its role in the meiosis and oocyte maturation regulation.

## RESULTS

### HDAC11 inhibition perturbs the meiotic maturation of porcine oocytes

**Figure 1:**
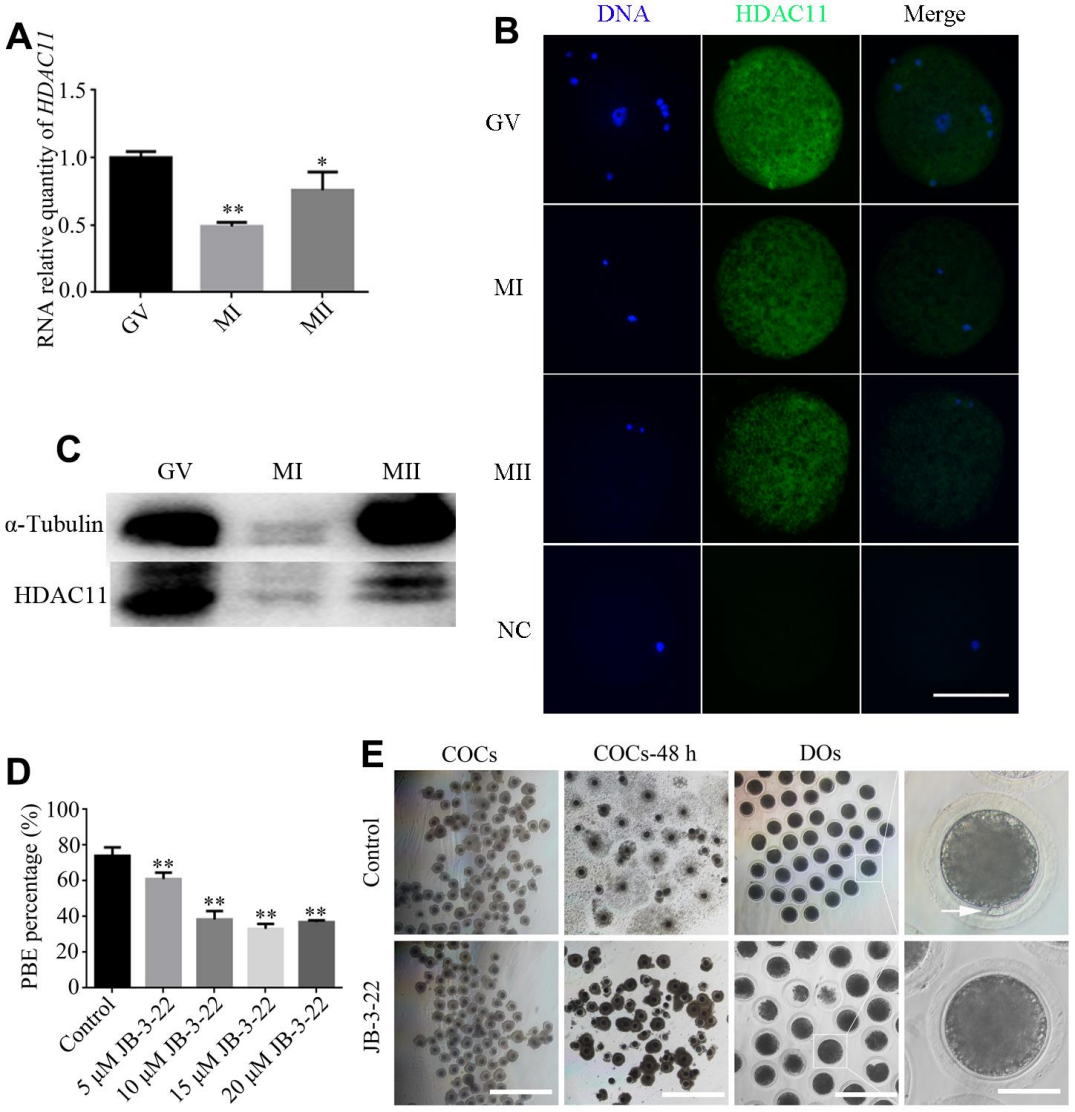
**Effects of different doses of JB-3-22 on the porcine oocyte maturation.** (**A**) mRNA expression of HDAC11 in porcine oocytes. GAPDH was used to normalize expression levels. The level of HDAC11 expression in GV oocytes was used as a calibrator (expression set to 1). (**B**) IF staining for HDAC11 protein in GV, MI and MII porcine oocytes. NC, negative control, MII oocyte stained with secondary antibody but not with primary antibody. Scale bar, 100 μm. (**C**) The results of Western blotting confirmed the expression of HDAC11 protein in porcine oocytes. (**D**) The rate of first polar extrusion (PBE) was recorded in control and different concentrations groups treated with JB-3-22 (5 μM, 10 μM, 15 μM, 20 μM) after culture for 44 h *in vitro*. (**E**) Typical image of oocyte maturation progression in control and JB-3-22 exposed oocytes. Cumulus cell expansion of oocyte complexes (COCs) and the PBE of denuded oocytes (DOs). Scale bar, 500 μm, 200 μm and 100 μm. The results represent the mean ± standard deviation of three independent experiments. * P < 0.05, and ** P < 0.01.

Firstly, we collected and analyzed *HDAC11* mRNA expression and protein level in porcine oocytes at different stages during *in vitro* maturation. Our results showed a dynamic expression profile for *HDAC11* mRNA and its expression at MI and MII stages was significantly lower than that at GV stage (P < 0.01) (Figure 1A). IF staining and WB results showed that HDAC11 protein was presented in porcine oocytes (Figure 1B, 1C). Furthermore, HDAC11 specific inhibitor, JB-3-22 (5 μM, 10 μM, 15 μM, 20 μM) was used to inhibit the expression of HDAC11 and to analyze the roles of HDAC11 on porcine oocytes maturation. As shown in Figure 1D, JB-3-22 treatment for 44 hours dramatically decreased porcine oocytes maturation rate in an dose dependent manner (control: 74.1 ± 1.4%, n = 200, 5 μM: 61.0 ± 2.2%, n = 200, 10 μM: 38.4 ± 2.1%, n = 200, 15 μM: 32.8 ± 1.1%, n = 200, 20 μM: 36.4 ± 0.6%, n = 200, P < 0.01). According to the result, 10 μM JB-3-22 was chose for the following experiments. JB-3-22 treatment resulted in oocyte meiotic arrest evidenced by the poor expansion of cumulus cells surrounding the COCs and decreased number of MII oocytes (Figure 1E). FT895, another HDAC11 inhibitor, also significantly decreased porcine oocytes maturation rate (Supplementary Figure 1). Altogether, our results indicated that HDAC11 might paly vital role in porcine oocytes maturation.

### HDAC11 inhibition caused aberrant spindle assembly and chromosome alignment during porcine oocytes maturation

**Figure 2:**
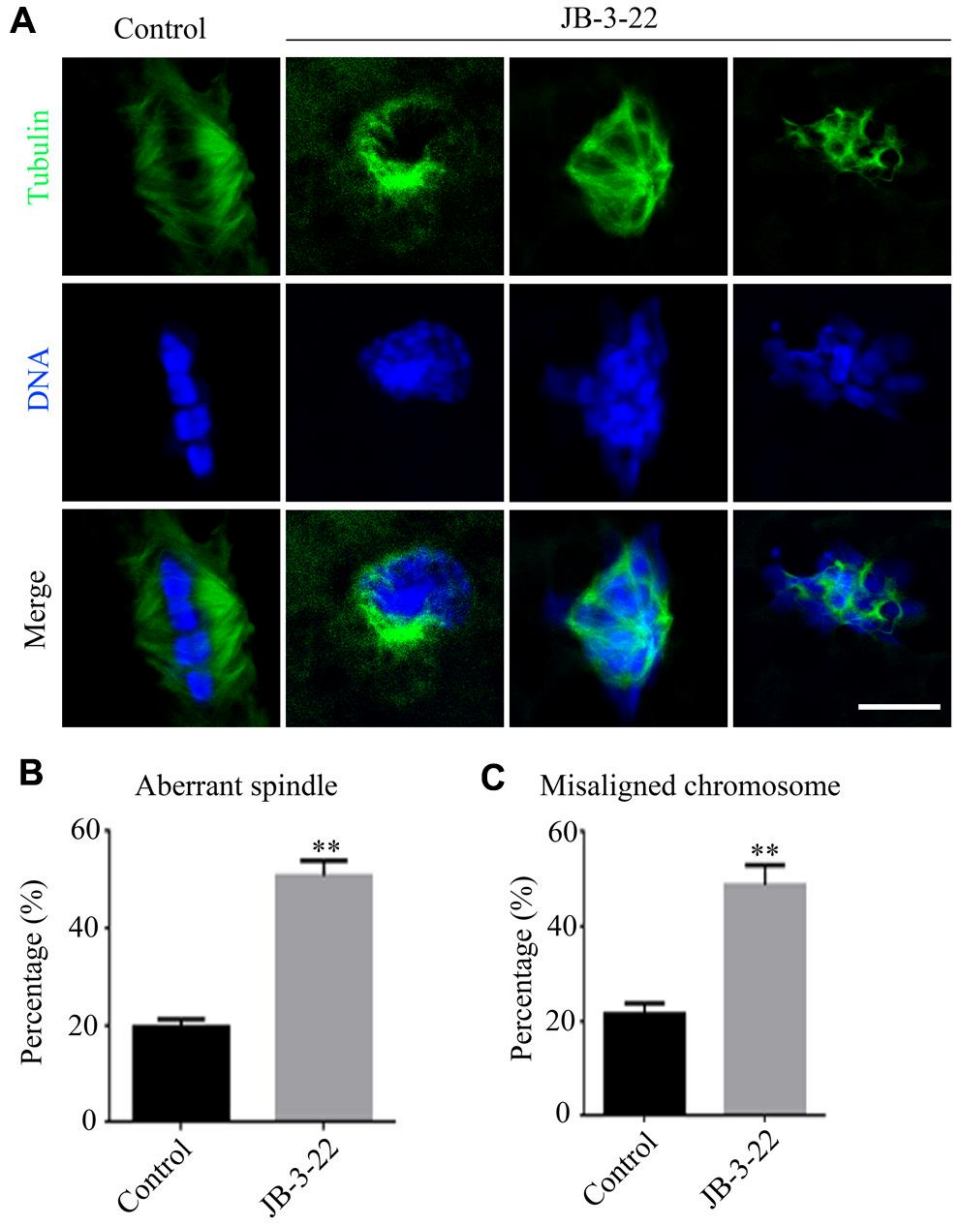
**JB-3-22 treatment caused abnormal spindle assembly and misaligned chromosome in porcine oocytes.** (**A**) Images of spindle morphologies and chromosome alignment in control and JB-3-22 treated oocytes, Scale bar, 5 μm. (**B**) The proportion of abnormal spindles was recorded in control and JB-3-22 treated oocytes. (**C**) The proportion of misaligned chromosomes was recorded in control and JB-3-22 treated oocytes. Data were presented as mean percentage (mean ± SEM) of at least three independent experiments. * P < 0.05 and ** P < 0.01.

Accurate spindle assembly and chromosome alignment are crucial events in the process of meiosis [15] which are hallmarks for the porcine oocyte maturation. Given that JB-3-22 treatment reduced porcine oocyte maturation rate, we sought to understand the role of HDAC11 inhibition by JB-3-22 treatment on the spindle assembly and chromosome alignment of porcine oocytes. The immune-labeled anti-α-tubulin antibody was used to show the spindle morphology, and nucleus was stained with Hoechst 33342 (10 μg/ml) to display chromosome alignment. The results showed that comparing with the control group, most oocytes in the JB-3-22 treated group showed aberrant spindle organization and chromosome arrangement (Figure 2A–2C). The proportion of oocytes with abnormal spindle morphology (50.8 ± 1.4%, n = 96) in JB-3-22 group was significantly higher than that in control group (19.7 ± 0.8%, n = 94, P < 0.01) (Figure 2B). Similarly, the proportion of oocytes with misaligned chromosome (48.8 ± 1.9%, n = 92) in JB-3-22 group was significantly higher than that in control group (21.6 ± 1.0%, n = 89, P < 0.01) (Figure 2C). FT895 treatment also caused aberrant spindle assembly and chromosome alignment during porcine oocytes maturation (Supplementary Figure 2).

### JB-3-22 treatment elevated the acetylation level of α-tubulin in porcine oocytes

**Figure 3:**
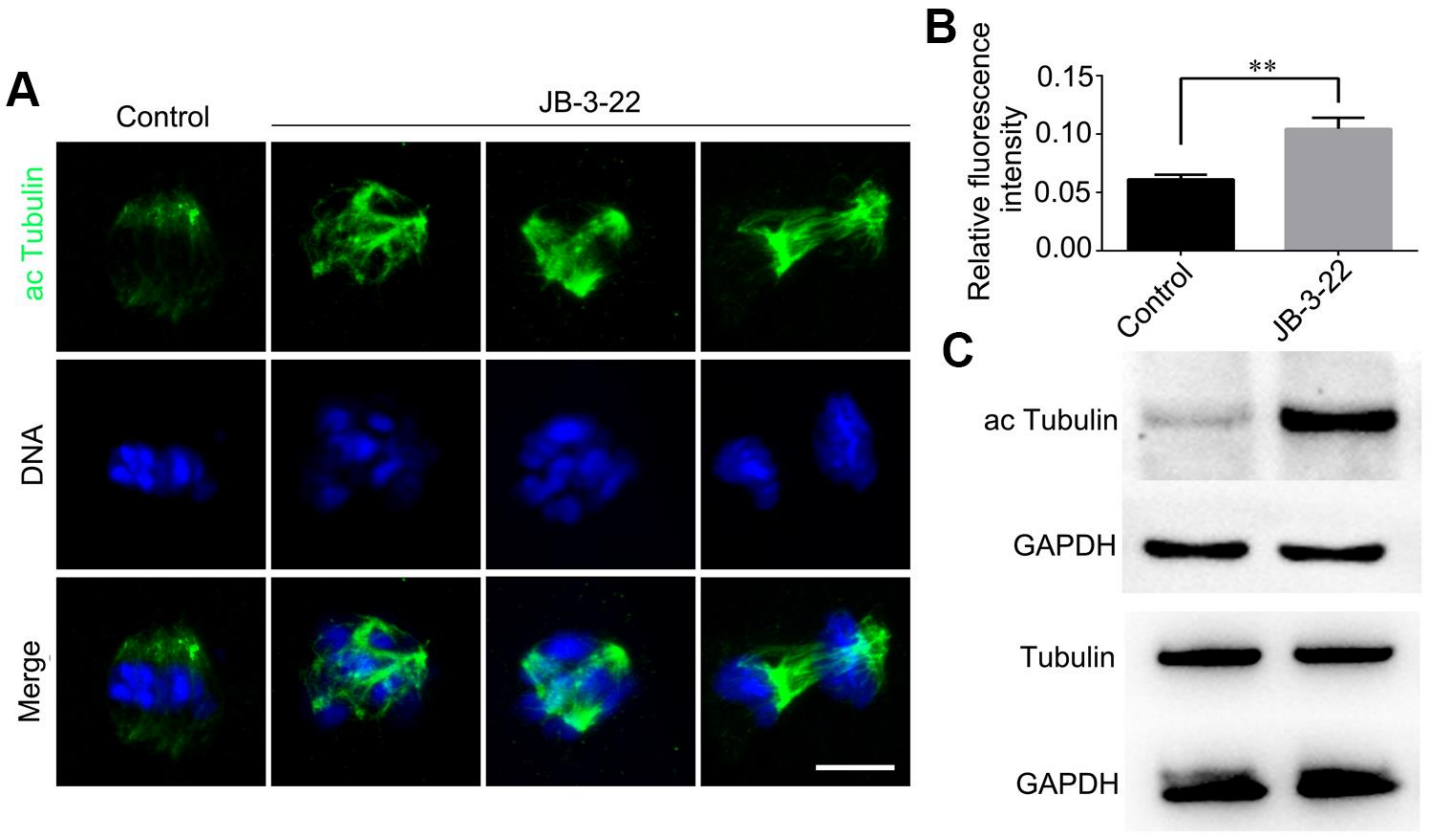
**JB-3-22 treatment elevated the acetylation level of α-Tubulin in porcine oocytes.** (**A**) Images of acetylated α-tubulin in control and JB-3-22-treated oocytes. Scale bar, 5 μm. (**B**) The fluorescence intensity of α-tubulin acetylation in control and JB-3-22 treated oocytes. The results represent the mean ± standard deviation of three independent experiments. ** P < 0.01. (**C**) The results of Western blotting confirmed the protein levels of acetylated tubulin in control and JB-3-22-treated oocytes.

HDAC11 is reported to be associated with HDAC6 in both the nucleus and cytoplasm [11]. HDAC6 could inhibit α-tubulin acetylation modification to affect spindle assembly and asymmetric division during mice oocytes maturation [6, 16]. Meanwhile, tubulin acetylation has been reported to regulate microtubules stability and functions [17, 18] which is important for spindle assembly and asymmetric division. So, we wondered whether the aberrant spindle assembly and chromosome misalignment in oocytes caused by inhibition of HDAC11 were a consequence of increased level of α-tubulin acetylation. The results from both the Western blotting and immunofluorescence staining (control: 0.105 ± 0.004, n = 48; JB-3-22: 0.061 ± 0.002, n = 56, P < 0.01; Figure 3A, 3B) showed that JB-3-22 treatment significantly increased α-tubulin acetylation level in porcine oocytes (Figure 3).

### JB-3-22 treatment elevated the H4 lysine acetylation in porcine oocytes

**Figure 4:**
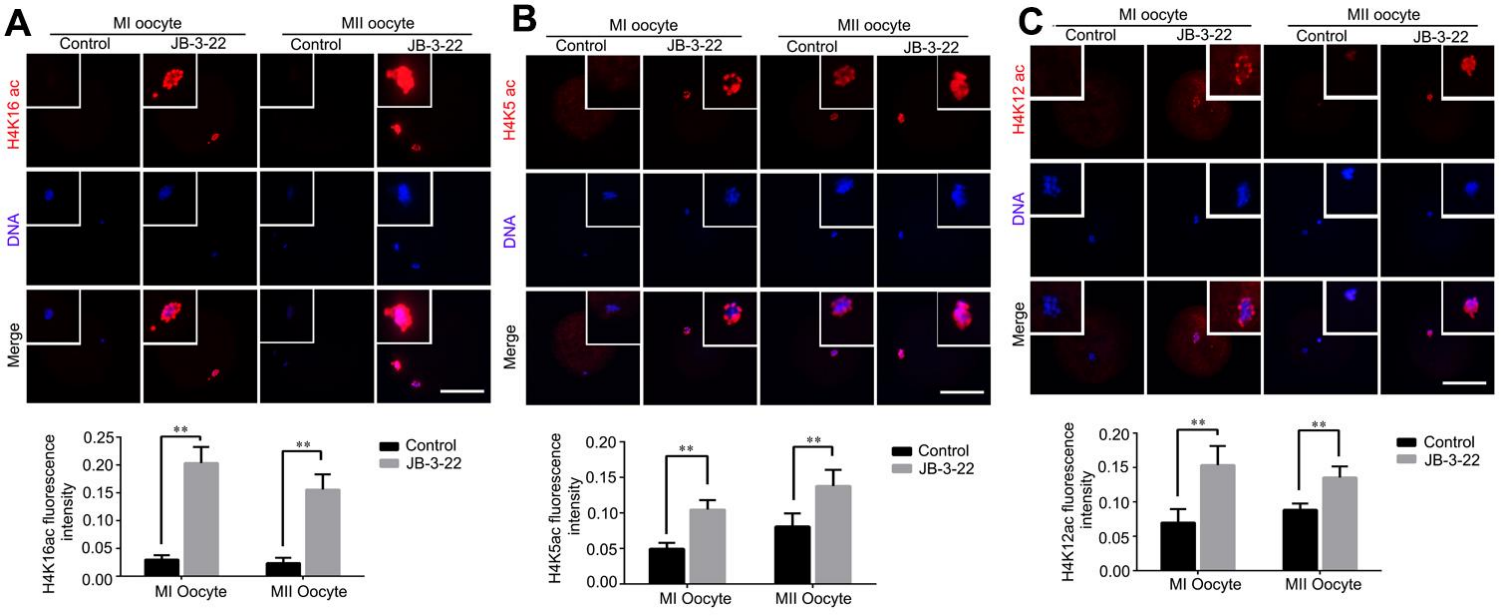
**JB-3-22 treatment elevated the acetylation levels of histone H4 lysine sites in porcine oocytes.** (**A**) Images of acetylated H4K16 in control and JB-3-22-treated oocytes at MI /MII stages. (**B**) Images of acetylated H4K5 in control and JB-3-22-treated oocytes at MI /MII stages. (**C**) Images of acetylated H4K12 in control and JB-3-22 treated oocytes at MI /MII stages. The results represent the mean ± standard deviation of three independent experiments. ** P < 0.01. Scale bar, 100 μm.

Given that the lysine acetylation modifications in H4 histone have been reported to play important roles in chromosomes alignment and separation during oocytes maturation, such as H4K16 acetylation [2], H4K5 acetylation and H4K12 acetylation [19]. Therefore, H4K5, H4K12 and H4K16 acetylation levels were analyzed by immunofluorescence staining in porcine oocytes after JB-3-22 treatment. The results showed that the acetylation levels of H4K16 (control MI: 0.204 ± 0.012, n = 52; JB-3-22 MI: 0.030 ± 0.003, n = 54, P < 0.01; control MII: 0.156 ± 0.010, n = 44; JB-3-22 MII: 0.024 ± 0.004, n = 50, P < 0.01; Figure 4A), H4K5(control MI: 0.105 ± 0.007, n =51; JB-3-22 MI: 0.050 ± 0.004, n = 52, P < 0.01; control MII: 0.138 ± 0.010, n = 46; JB-3-22 MII: 0.081 ± 0.008, n = 50, P < 0.01; Figure 4B) and H4K12 (control MI: 0.154 ± 0.011, n = 57; JB-3-22 MI: 0.070 ± 0.007, n = 49, P < 0.01; control MII: 0.136 ± 0.007, n = 46; JB-3-22 MII: 0.089 ± 0.003, n = 52, P < 0.01; Figure 4C) in JB-3-22 treated MI and MII oocytes were significantly higher than those in control group. FT895 treatment also significantly increased the acetylation level of H4K16 in porcine MII oocytes (Supplementary Figure 3).

### JB-3-22 treatment regulates phosphorylation status of histone H3 in porcine oocytes

**Figure 5:**
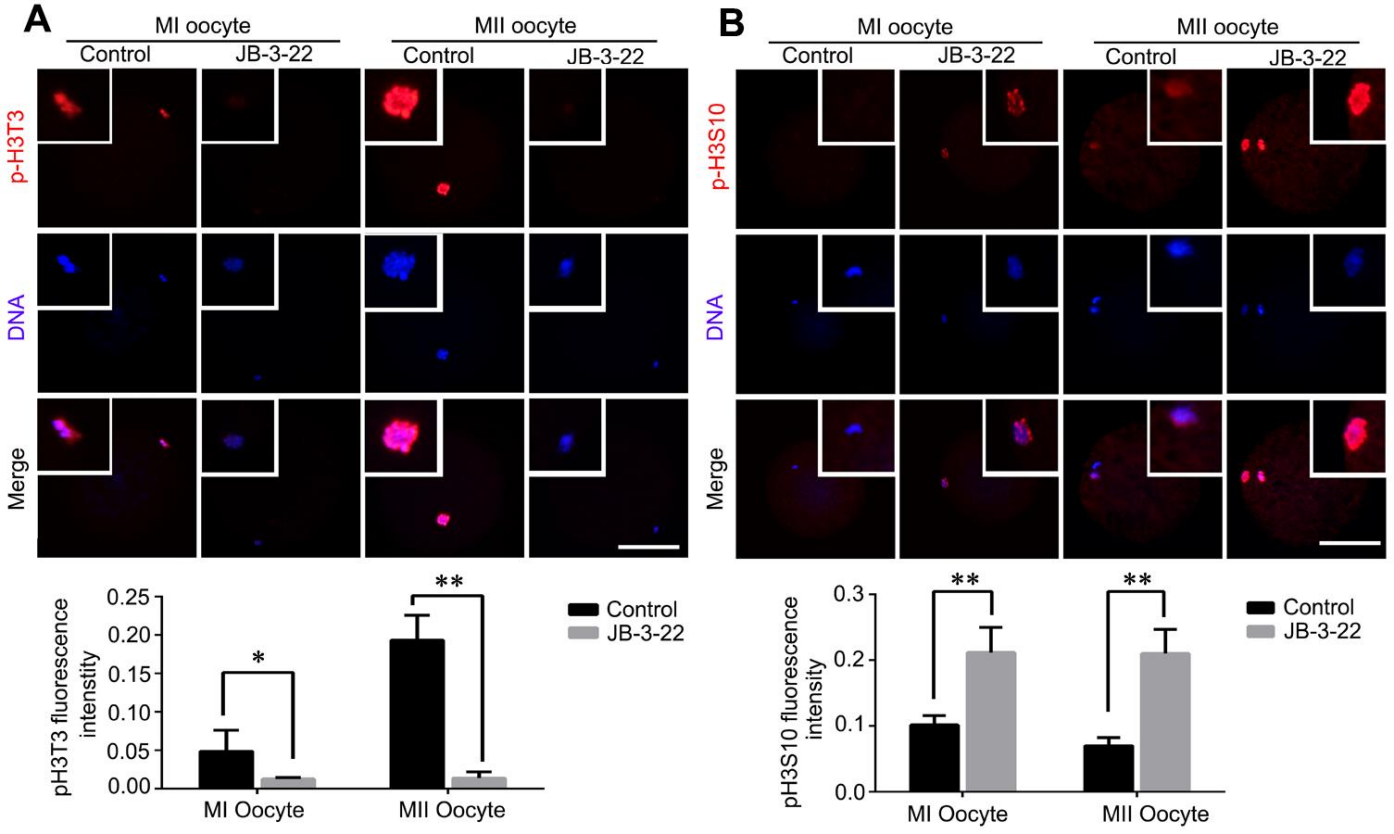
**JB-3-22 treatment increased the phosphorylation level of H3S10 and reduced the phosphorylation level of H3T3 in porcine oocytes.** (**A**) Images of phosphorylated H3T3 in control and JB-3-22-treated oocytes at MI and MII stages. (**B**) Images of phosphorylated H3S10 in control and JB-3-22-treated oocytes at MI and MII stages. The results represent the mean ± standard deviation of three independent experiments. * P < 0.05 and ** P < 0.01. Scale bar, 100 μm.

It was reported that there were cross-talking among H4K14 acetylation, H3T3 phosphorylation and H3S10 phosphorylation [20]. Moreover, the phosphorylation status of H3T3 and H3S10 plays important roles in regulating mice oocytes maturation [21]. The increased acetylation of H4K16 warrants us to examine the changes of H3T3 and H3S10 phosphorylation in porcine oocytes treated with JB-3-22. Interestingly, our results showed that H3T3 phosphorylation level was significantly decreased in porcine oocytes upon JB-3-22 treatment (control MI: 0.013 ± 0.001, n = 42; JB-3-22 MI: 0.049 ± 0.014, n = 49, P < 0.05; control MII: 0.014 ± 0.003, n = 43; JB-3-22 MII: 0.193 ± 0.010, n = 41; P < 0.01) (Figure 5A). In contrast, the H3S10 phosphorylation level was significantly increased in porcine oocytes upon JB-3-22 treatment (control MI: 0.212 ± 0.014, n = 43; JB-3-22 MI: 0.102 ± 0.005, n = 48, P < 0.01; control MII: 0.210 ± 0.018, n = 53; JB-3-22 MII: 0.070 ± 0.007, n = 54; P < 0.01) (Figure 5B).

### JB-3-22 treatment had no significant effect on cytoplasmic maturation

**Figure 6:**
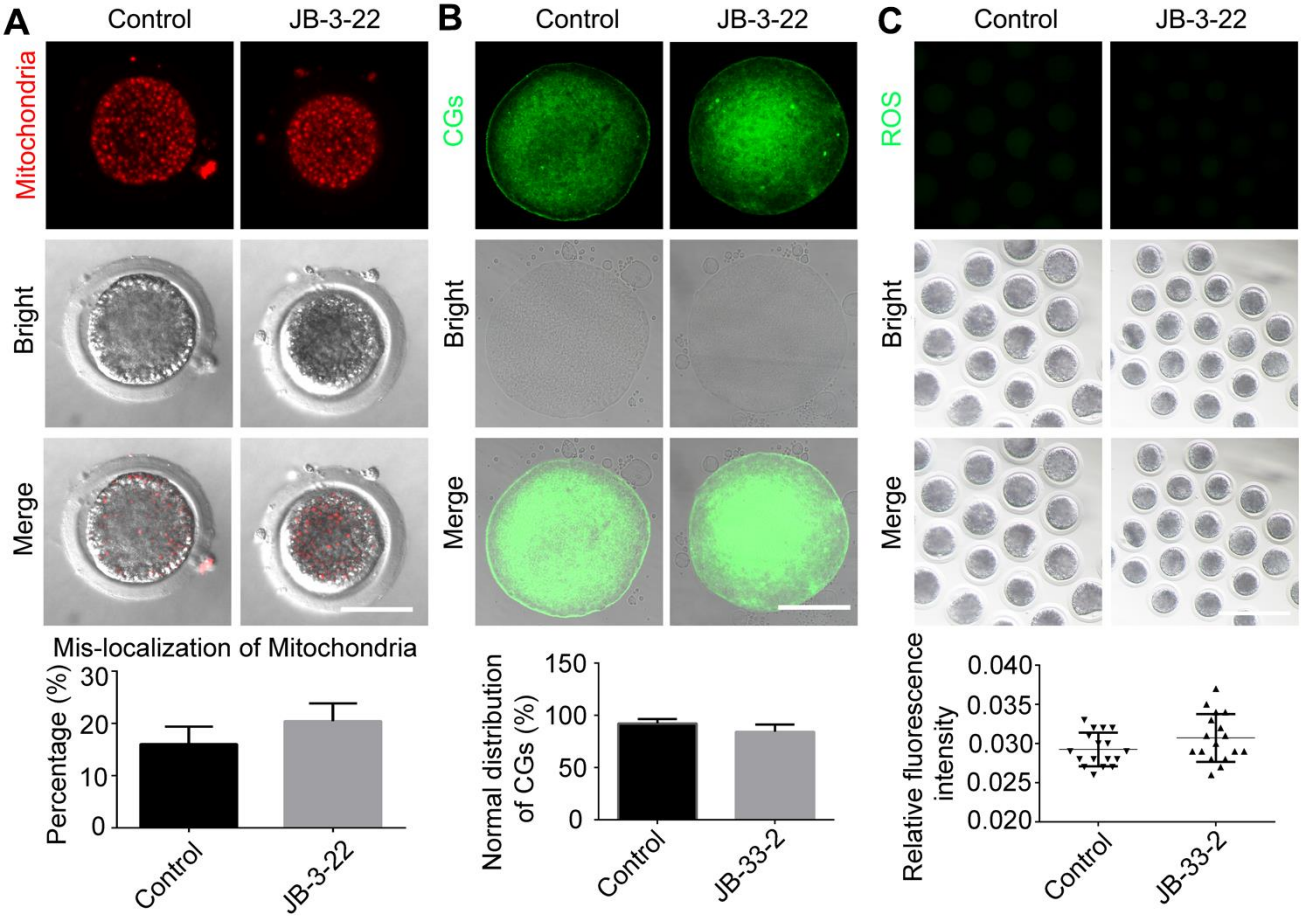
**JB-3-22 treatment had no significant effect on the cytoplasm maturation process of porcine oocytes.** (**A**) Images of distribution of mitochondria in control and JB-3-22-treated oocytes. Scale bar, 100 μm. (**B**) Images of migration of cortical granules in control and JB-3-22-treated oocytes. Scale bar, 100 μm. (**C**) Images of ROS level in control and JB-3-22-treated oocytes. Scale bar, 500 μm.

Nuclear maturation and cytoplasm maturation are important events for successful MII oocytes generation and maturation. Moreover, cytoplasm maturation, mainly including changes in organelle morphology and function, mRNA and protein accumulation and changes in cell metabolism, is more complex than nuclear maturation and not easy to be observed [22, 23]. Therefore, the aberrant distribution of mitochondria (control: 20.8 ± 1.5%, n = 38; JB-3-22: 16.5 ± 1.5%, n = 36, P > 0.05; Figure 6A), migration of cortical granules (CGs) (control: 84.4 ± 3.0%, n = 41; JB-3-22: 92.3 ± 1.8%, n = 46, P > 0.05; Figure 6B) and reactive oxygen species (ROS) content (control: 0.030 ± 0.001, n = 57; JB-3-22: 0.029 ± 0.001, n = 56, P > 0.05; Figure 6C) in porcine oocytes in control and JB-3-22 treated group were further analyzed. The results indicated that there was no statistical difference in cytoplasmic maturation between the two groups (Figure 6), which suggested that JB-3-22 treatment had no significant effect on the cytoplasm maturation of porcine oocytes during meiosis.

### HDAC11 inhibition disturbed the transcriptome in porcine oocytes

**Figure 7:**
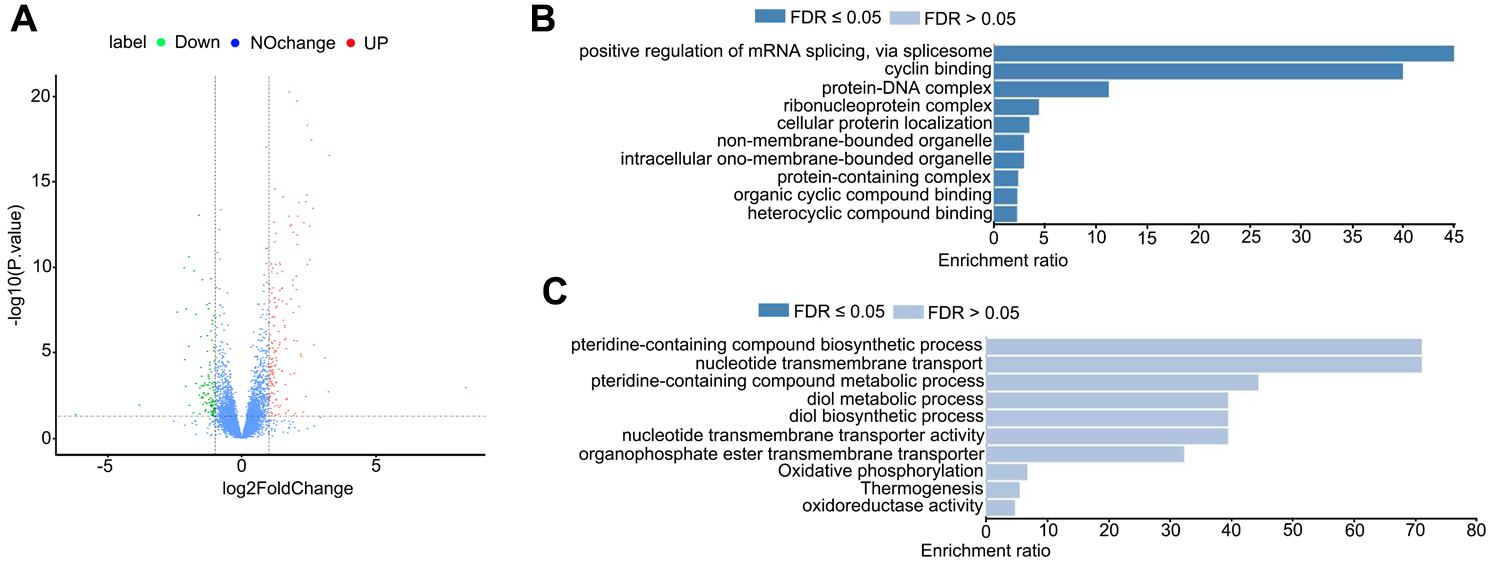
**JB-3-22 treatment disrupted the mRNA transcription in porcine oocytes.** (**A**) Volcano plot of differences in gene expression in JB-3-22 treated oocytes. Each point represents one gene. The x-axis represents the delta beta value (control and JB-3-22 group), and the y-axis indicates –log10 of the *p*-value. (**B**) The GO analysis of upregulated genes. (**C**) The GO analysis of downregulated genes.

Next, we sought to understand the influences of HDAC11 on the transcriptome of oocytes and the transcriptome sequencing (RNA-seq) was performed on control and JB-3-22 treated MII oocytes. Interestingly, the volcano plot showed that 157 genes were upregulated and 106 genes were downregulated in JB-3-22 treated oocytes (Figure 7A). GO analysis revealed that upregulated genes mainly associated with mRNA splicing and cyclin binding (Figure 7B), and the downregulated genes mainly associated with pteridine-containing compound biosynthetic process and nucleotide transmembrane transport (Figure 7C).

### JB-3-22 promoted the apoptosis of porcine oocytes and disrupted the embryonic development

**Figure 8:**
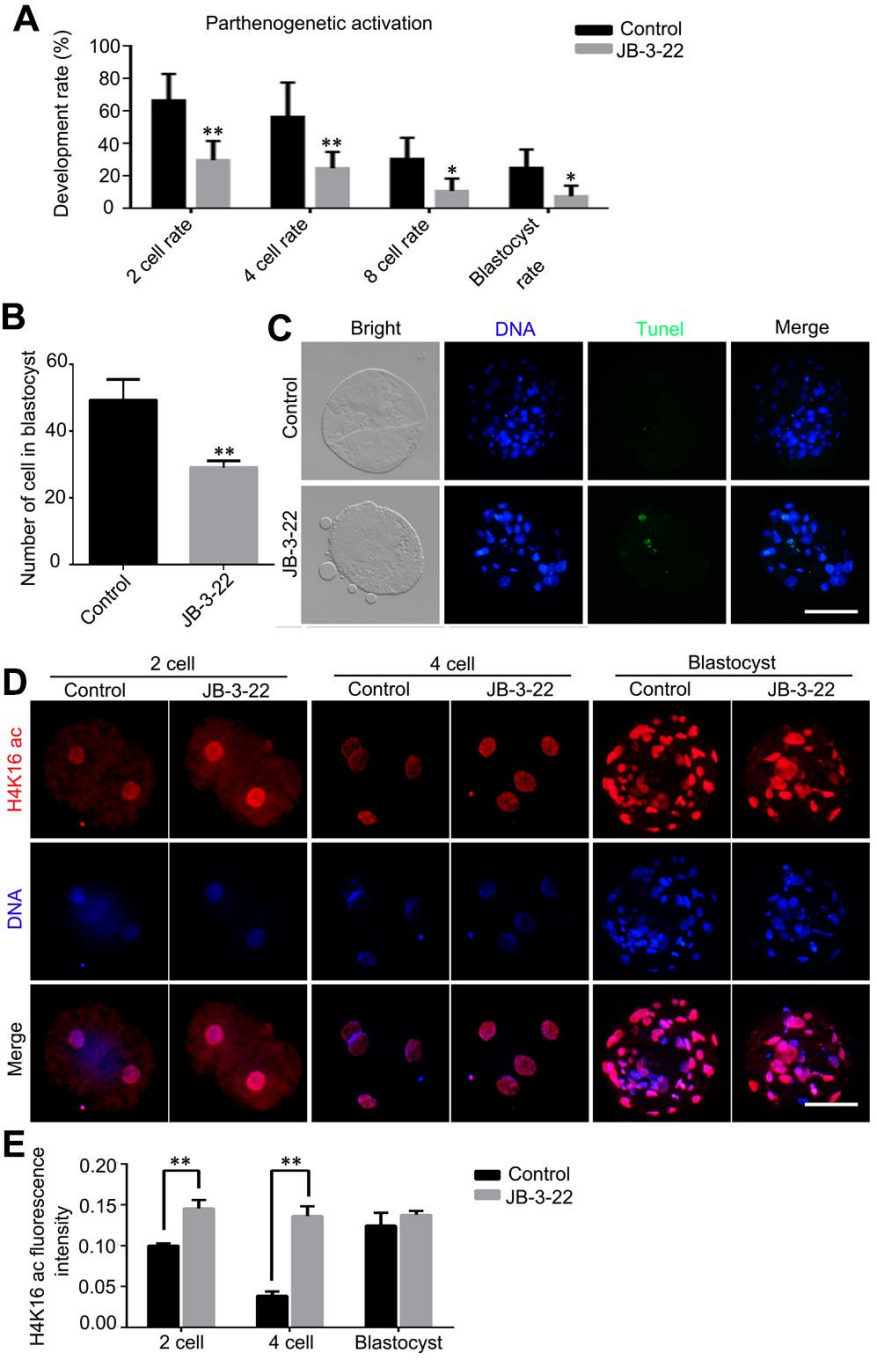
**JB-3-22 promoted the apoptosis of porcine oocytes and disrupted the subsequent embryonic development of porcine oocytes after parthenogenetic activation.** (**A**) The rates of different development stage embryos were recorded in control and JB-3-22 treated oocytes after parthenogenetic activation. (**B**) Images of apoptotic cells in blastocysts in control and JB-3-22 treated group respectively. (**C**) The statistics of total cell number in per blastocyst. (**D**) Images of H4K16 acetylation at different stages of parthenogenetic embryo development in control and JB-3-22 group. (**E**) The fluorescence intensity of H4K16 acetylation. The results represent the mean ± standard deviation of three independent experiments. * P < 0.05 and ** P < 0.01. Scale bar, 100 μm.

Given that some JB-3-22 treated oocytes bypass the inhibition of JB-3-22 treatment and physiologically matured to the MII phase, the development ability of those oocytes after parthenogenetic activation was further analyzed. The results showed that JB-3-22 treatment significantly decreased the cleavage rate (control: 66.60 ± 9.40, n = 165; JB-3-22: 29.97 ± 6.73, n = 157, P < 0.01), 4 cell rate (control: 56.57 ± 12.15, n = 165; JB-3-22: 24.87 ± 5.83, n = 157, P < 0.01), 8 cell rate (control: 30.60 ± 7.53, n = 165; JB-3-22: 10.93 ± 4.38, n = 157, P < 0.05) and blastocyst formation rate (control: 25.23 ± 6.42, n = 165; JB-3-22: 7.60 ± 3.81, n = 157, P < 0.05) (Figure 8A). JB-3-22 treatment also significantly decreased the total cell number of porcine parthenogenetic blastocysts (control: 49.2 ± 2.8, n = 15; JB-3-22: 29.0 ± 1.2, n = 12, P < 0.01) (Figure 8B), but increased the apoptotic cell proportion of blastocyst (Figure 8C). Furthermore, we sought to understand whether the parthenogenetic embryos still maintain the high level of H4K16 acetylation modification after JB-3-22 treatment. The result from immunofluorescence staining showed that the high H4K16 acetylation modification in JB-3-22 treated oocytes was persisted in 2 cells and 4 cells stage, but recovered to normal level at blastocysts stage (2 cell: control: 0.100 ± 0.002, n = 25; JB-3-22: 0.146 ± 0.005, n = 22, P < 0.01; 4cell: control: 0.039 ± 0.002, n = 23; JB-3-22: 0.136 ± 0.006, n = 22, P < 0.01; Blastocysts: control: 0.125± 0.007, n = 25; JB-3-22: 0.138 ± 0.002, n = 22, P > 0.05) (Figure 8D, 8E). Altogether, those results suggested that the effects of JB-3-22 treatment on histone acetylation can be lasted for a longer time until the 4 cells stage.

## DISCUSSION

Many HDACs members have been reported to play important roles during oocyte meiosis progress, such as HDAC2 [2], HDAC3 [3], HDAC6 [4] and HDAC8 [5]. More importantly, HDAC11 has been reported to be associated with HDAC6 in both the nucleus and cytoplasm [11]. Therefore, we proposed that like HDAC6, the HDAC11 might function during mammalian oocyte maturation. In the present study, we found that HDAC11 was expressed in porcine oocytes at different stages. Similar to the reported results for HDAC6 inhibition in mouse oocytes [24], the inhibition of HDAC11 by JB-3-22 significantly decreased maturation rate of porcine oocytes and subsequent development by regulating the histone acetylation, histone phosphorylation and transcriptome.

HDAC11 plays important role in spindle assembly and the cell cycle during mitosis of neuroblastoma cells [12]. Furthermore, aberrant spindle assembly and chromosome alignment could hamper the oocytes maturation [24]. Therefore, we sought to explore the effect of HDAC11 on porcine oocyte maturation and our data showed that HDAC11 inhibition disturbed spindle assembly and chromosome alignment during porcine oocytes maturation. Those remarkable phenotypes warranted us to explore the underlying mechanisms. Spindle is composed of microtubule which stability and function could be regulated by tubulin acetylation [25]. Histone acetyltransferases [26] and class I and II HDACs [3, 24] have been reported to inhibit tubulin acetylation and deacetylation and play important roles on spindle organization during mice oocytes meiosis. Moreover, the catalytic core region of HDAC11 showed sequence similarity with class I and II HDACs at amino acid level [8]. Above data implied that HDAC11 may also activate tubulin deacetylation and then ensure spindle organization during oocytes meiosis. In line with this, HDAC11 inhibition by JB-3-22 treatment significantly increased the tubulin acetylation level during porcine oocytes meiosis, as did in mice oocytes after HDAC6 [24] or HDAC3 [3] inhibition.

Except for spindle, kinetochore also plays important roles on chromosome alignment and segregation during meiosis progress. Kinetochore is a protein complex of nearly 100 proteins that connects centromere DNA with spindle microtubules and thereby couples with the forces generated by microtubule dynamics to provide power for chromosomal motions [27, 28]. Moreover, the acetylation of histone H4 lysine, such as H4K16 acetylation [29], H4K5 acetylation and H4K12 acetylation [19], could regulate the formation of kinetochore in DT40 cells. The acetylation of histone H4 occurs first at lysine 16 (K16), then at K8 or K12, and ultimately at K5 [30]. Inhibited Esco2 [29], HDAC2 [2] or HDAC6 [24] activity could disturb H4K16 acetylation, then destroyed the attachment between kinetochores and microtubules (K-MT) in mice oocytes. In this study, we found HDAC11 inhibition also significantly increased H4K16, H4K5 and H4K12 acetylation. Except for histone H4 lysine acetylation, many other histone modifications have also been reported to be essential for oocytes maturation, such as H3T3 [21] and H3S10 [31] phosphorylation. More importantly, it was reported that there were cross-talking among H4K16 acetylation, H3T3 phosphorylation and H3S10 phosphorylation [20]. In mice oocytes, the phosphorylation of H3T3 and H3S10 were decreased following the elevation of H4K16 acetylation upon HDAC6 inhibition [24]. However, our results showed that H3T3 phosphorylation level was decreased but H3S10 phosphorylation level was increased following the elevation of H4K16 acetylation caused by HDAC11 inhibition in porcine oocytes. Those data implied that HDAC11 may also play important roles on K-MT stability and the pulling forces across kinetochores as HDAC2 [2] and HDAC6 [24] did in mice oocytes, which may contribute to the spindle disorganization and chromosome alignment failure in porcine oocytes.

Cytoplasmic maturation is an important index for oocytes maturation, which includes the changes in organelle morphology and function, mRNA and protein accumulation and cell metabolism [22, 23]. Therefore, we investigated the roles of HDAC11 on cytoplasmic maturation in porcine oocytes and our results showed that HDAC11 inhibition did not disturb the mitochondria distribution, cortical granules migration and ROS content in porcine oocytes. However, HDAC11 inhibition affected the transcriptome of porcine oocytes and 157 genes were upregulated and 106 genes were downregulated in porcine oocytes. The changed transcriptome may result from two ways, transcription activity change or mRNA stability change which need further study. Moreover, HDAC11 inhibition compromised the development ability of MII oocytes after parthenogenetic activation, which may be caused by the aberrant nuclear maturation and mRNA accumulation.

In conclusion, our study indicated that HDAC11 is involved in meiotic spindle formation, chromosome alignment and segregation and mRNA transcription during porcine oocytes maturation by regulating α-tubulin acetylation and histone modifications.

## MATERIALS AND METHODS

### Chemicals and reagents

All chemicals and reagents were purchased from Sigma (St. Louis, MO, USA) unless otherwise noted.

### Collection and *in vitro* maturation of porcine oocytes

Porcine ovaries were collected from a local abattoir and transported to the laboratory within 2-4 h in 0.9% sodium chloride at 35-38.5° C with penicillin. Cumulus oocytes complexes (COCs) were extracted by a 10 ml syringe from 3-6 mm ovarian follicles on the surface of the ovary. COCs with at least three layers of cumulus cells were selected and washed twice with the PBS supplemented with 10% fetal bovine serum. Then the COCs were cultured *in vitro* mature medium [32]. COCs were cultured in the mature medium I which covered by liquid paraffin oil in the incubator at 38.5° C with 5% CO_2_ for 22-24 h, and then cultured in mature medium II until 42-44 h. Oocytes with first polar body were considered to be mature oocytes. MI (Metaphase I) and MII (Metaphase II) oocytes were collected at 27 h and 42 h after maturation *in vitro*, respectively.

The mature medium I was composed of TCM-199 supplemented with 26 mM sodium bicarbonate, 3.1 mM glucose, 0.9 mM sodium pyruvate, 10 μg/ml epidermal growth factor, 50 μg/ml luteinizing hormone, 50 μg/ml follicle-stimulating hormone, 0.1% polyvinyl alcohol (PVA), 0.03% bovine serum albumin (BSA), and 0.1% penicillin/streptomycin (Gibco), while the mature medium II was the same with mature medium I without hormones.

### Immunofluorescence staining and confocal microscopy analysis

After removing the zona pellucida by acidic Tyrode solution (pH 2.5), the porcine oocytes were fixed in darkness for 30 min with 4% paraformaldehyde, and permeabilized by 1% Triton X-100/PBS (Phosphate-buffered saline) (v/v) for 20 min. Following permeabilization, the oocytes were blocked with PBS containing 1% bovine serum albumin (BSA) at 37° C for 1 h, porcine oocytes were incubated with primary antibodies (Table 1) at 4° C overnight. Then, the oocytes were incubated with secondary antibodies at 37° C for 1.5 h, oocyte nucleus were stained with Hoechst 33342 (10 μg/ml) for 15 min. The primary antibodies and secondary antibodies were diluted by PBS with 0.1% BSA. Oocytes were washed with PBS three times between each step above. Prolong Gold Antifade Mountant (Invitrogen, P36930) was dropped in the center of the slide and the oocytes after the above treatment were transferred to the drop followed by the coverslip on it. Fluorescence was detected by a fluorescence microscope (Nikon, Tokyo, Japan) or confocal microscope (LSM 8800, Zeiss, Oberkochen, Germany).

**Table 1:** Antibodies used for immunofluorescence staining and WB.

**Antibodies**	**Host species**	**Catalog number**	**Manufacturer**	**Dilution ratio**
H4K16ac	Rabbit	ab109463	Abcam	1:200
H4K5 ac	Rabbit	AF1219	Beyotime	1:200
H4K12ac	Rabbit	ab61238	Abcam	1:200
p-H3T3	Rabbit	ab78351	Abcam	1:200
p-H3S10	Rabbit	9701S	Cell Signaling Technology	1:200
Goat anti Rabbit 594	Rabbit	S0006	affinity	1:200
Ac-α-Tubulin	Rabbit	#5335	Cell Signaling Technology	1:200 and 1:1000
α-Tubulin-FITC	Mouse	F2168	Sigma	1:200
α-Tubulin	Mouse	ab7291	Abcam	1:3000
GAPDH	Mouse	60004-1-Ig	Proteintech	1:1000

### Extraction of RNA and quantitative PCR

Total RNA was extracted from 200 porcine oocytes without zona pellucida by RNeasy Mini kit (Qiagen, Hilden, Germany), and cDNA was synthesis with TransScript All-in-One First-Strand cDNA (TranGen Biotech, Beijing, China), each 20 μl reaction system contained 6 μl total RNA, 1 μl gDNA Remover, 4 μl 5×TransScript All-in-One SuperMix for qPCR (including RNase Inhibitor, Anchored Oligo (dT) 18 Primer, RandomPrimer (N9), dNTPs and buffer) and 9 μl RNase-free Water. The cDNA amplification conditions were as follows: 45° C for 15 min and 85° C for 15s. qPCR was performed in the 96-well plate. Each 20 μl reaction system contained 10 μl SYBR green premix, 0.5 μl forward and 0.5 μl reverse primers (Table 2) (10 μM), 1 μl cDNA and 8 μl non-enzymatic water. The amplification conditions were as follows: denaturation for 30 s at 95° C, 40 cycles of PCR (5 s at 95° C and 30 s at 60° C), The melt-curve was 5 s at 95° C, 60 s at 60° C and 1 s at 95° C, and then hold at 4° C. The relative expression of each gene was calculated using 2^-ΔΔCT^ method.

**Table 2:** Primer used for quantitative reverse-transcription PCR.

**Gene**	**Forward primer**	**Reverse primer**
HDAC11	AAGCCTCCAGGTGCCCATCC	GCGAGTCTGAGTTCTGTGCTGAG
GAPDH	GTGTCCTGTGACTTCAAC	CTGTAGCCAAATTCATTGTC

### Western blotting analysis

After removing the zona pellucida, 200 porcine oocytes were transferred into 20 μl RIPA buffer (Beyotime, China) which containing 1% protease inhibitor, then added 4×SDS loading buffer and boiled at 100° C for 5 min. Total proteins were subjected to electrophoresis on Meilun 10% SDS-PAGE gel by constant voltage of 135 V for 45 min with each hole about 20 μl sample weight, then the protein was transferred to PVDF membrane by 400 mA for 35 min, and after blocking by TBST containing 5% skimmed milk powder for 2 h, the membranes were incubated with primary antibodies (Table 1) (1:1000) at 4° C overnight. Next day, the membranes were incubated with HRP-conjugated secondary antibodies at room temperature for 2 h. After been washing three times by TBST, the membranes were covered by ECL (Thermo, USA) and protein bands were detected by Tanon-3900 (Tanon, China). GAPDH was used as internal reference protein.

### Parthenogenetic activation

Porcine oocytes with the first polar body were put in the fluid of fusion (0.3 M mannitol, 1 mM CaCl_2_ 2H_2_O, 1 mM MgCl_2_ 6H_2_O and 0.5 mM HEPES) for a few seconds, and then into the electrical activation slot followed a linear one-way permutation between the positive and negative electrode. Activated for two consecutive direct-current pulses at 1.2 kV/cm for 30 μs by an ECM2001 electro-fusion instrument (ECM2001, BTX, USA). After activation, the porcine oocytes were transferred into the embryo culture fluid, PZM, and then cultured at 38.5° C with 5% CO_2_ for 7 days to develop to the blastocyst stage.

### Detection of blastocyst apoptosis

After the removal of zona pellucida, fixation by 4% paraformaldehyde, permeabilization by 1% Triton-X 100 and blocked by 1% BSA, the blastocysts were incubated with term deoxynucleotidyltransferase dUTP nick end labelling (TUNEL) solution from the *In Situ* Cell Death Detection Kit (Roche, Mannheim, Germany) at 37° C for 1 h in darkness. After being washed with PBS for 3 times, the nuclei were restained with Hoechst 33342 (10 μg/ml) for 15 min. The stained blastocysts were mounted between a cover slip and a glass slide, and observed under a fluorescence microscope.

### RNA preparation and RNA-seq

Smart-Seq2 method was used to amplify each samples (control and 10 μg/ml JB-3-22 treated MII oocytes, ten oocytes for each group) according the manufacturer^’^s instruction. RNA concentration of library was measured by Qubit 2.0 Flurometer (Life Technologies, CA, USA). Agilent Bioanalyzer 2100 system was used to assess the insert size and the quality of the amplified products was evaluated according the detection results. Amplified product cDNA was used as input for the library construction of transcriptome. After the library construction, Agilent Bioanalyzer 2100 system was used to assess insertion size, and Taq-Man fluorescence probe of an AB Step One Plus Real-Time PCR system (Library valid concentration > 10 nM) was used to quantify the accurate insertion size. Clustering of the index-coded samples was performed using a cBot cluster generation system and the HiSeq PE Cluster Kit v4-cBot-HS (Illumina). Then, the libraries were sequenced by Zhejiang Annoroad Biotechnology (Beijing, China) on an Illumina platform, and 150 bp paired-end reads were generated. The estimated transcript counts from RNA-seq were first normalized using TMM normalization and were transformed using the voom method. Differentially expressed genes of RNA-seq were identified based on a false discovery rate of < 0.05 and estimated absolute log2 fold change > 1 between different genotypes.

### Statistical analysis

Data were analyzed by paired-sample t-test or ordinary one-way ANOVA test. All experiments were repeated at least three times, P < 0.05 was considered statistically significant; P < 0.01 was considered extremely significant.

## Supplementary Material

Supplementary Figures
