# Supplementary material for: HDAC11 inhibition disrupts porcine oocyte meiosis via regulating α-tubulin acetylation and histone modifications
Source: Aging (Albany NY). 2021 Mar 19;13(6):8849–64. doi: 10.18632/aging.202697 (PMC8034937; doi:10.18632/aging.202697)
Supplement: Supplementary Figures [file aging-13-202697-s001.pdf]

SUPPLEMENTARY FIGURES

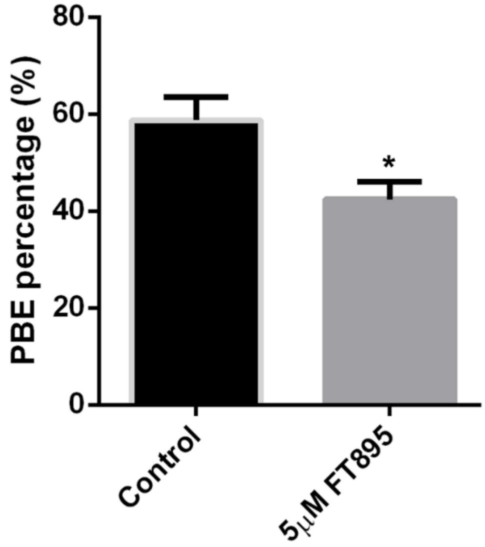

Supplementary Figure 1. The rate of first polar body extrusion (PBE) in control and FT895 treated groups after culture for 44 h *in vitro*. The results represent the mean ± standard deviation of three independent experiments. * $P < 0.05$.

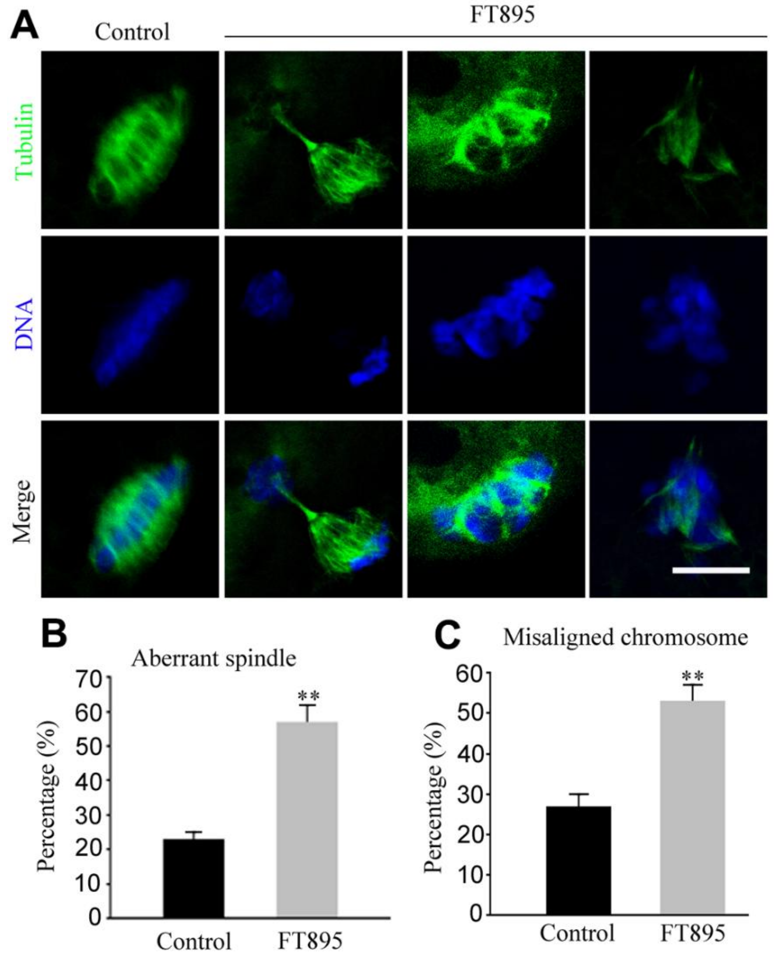

Supplementary Figure 2. (**A**) Images of spindle morphologies and chromosome alignment in control and FT895 treated oocytes, Scale bar, 5 µm. (**B**) The proportion of abnormal spindles was recorded in control and FT895 treated oocytes. (**C**) The proportion of misaligned chromosomes was recorded in control and FT895 treated oocytes. Data were presented as mean percentage (mean ± SEM) of at least three independent experiments. ** P < 0.01.

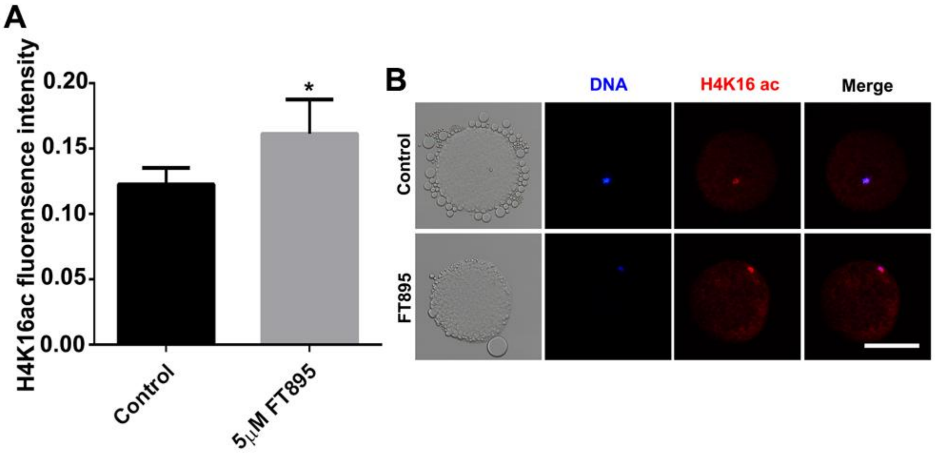

Supplementary Figure 3. (**A**) The fluorescence intensity of H4K16 acetylation in control and FT895 treated oocytes. (**B**) Images of acetylated H4K16 in control and FT895 treated oocytes at MII stages, Scale bar, 100 µm. The results represent the mean ± standard deviation of three independent experiments. * P < 0.05.
